# Difficulties with positive, but not negative, emotion regulation moderate the association between positive alcohol expectancies and alcohol use in college students

**DOI:** 10.1016/j.abrep.2025.100583

**Published:** 2025-01-07

**Authors:** Katherine Shircliff, Haley Coronado, Madeline McClinchie, Caroline Cummings

**Affiliations:** Texas Tech University Department of Psychological Sciences United States

**Keywords:** Affect, Alcohol use, College students, Emotion dysregulation

## Abstract

**Background:**

Positive alcohol expectancies are linked to increased alcohol use among college students. Difficulties regulating emotion have been shown to moderate this relationship, though little research accounts for differences based on the valence of the emotion being regulated.

**Objective:**

To examine the independent moderating roles of positive and negative emotion dysregulation on the association between positive alcohol expectancies and alcohol use.

**Methods:**

College students (N = 165, M_age_ = 20.48, SD_age_ = 1.90; 66.1 % Female; 66.7 % White; 65.5 % non-Hispanic) who reported regular substance use (≥ 3 times in the past week) completed a one-time survey. Linear regression analyses with moderation were conducted.

**Results:**

Correlational analyses suggested that positive alcohol expectancies, positive emotion dysregulation, and negative emotion dysregulation were positively associated with greater alcohol use. Linear regression analyses indicated that difficulties with positive emotion regulation moderated the relationship between positive alcohol expectancies and alcohol use. However, difficulties with negative emotion regulation did not moderate this relationship. College students who reported greater positive alcohol expectancies and concurrently greater difficulties with positive emotion regulation also report greater alcohol consumption, compared to those who reported greater positive alcohol expectancies and fewer difficulties regulating positive emotion.

**Discussion:**

Difficulties with positive, not negative, emotion regulation may serve as a risk factor for hazardous alcohol use in college students. Findings may inform the modification of existing intervention programs across university counseling centers and other health sectors to promote the development of positive emotion regulation skills for individuals who endorse positive emotion regulation difficulties, thereby reducing hazardous alcohol use amid this high-risk developmental period.

## Highlights

• Positive alcohol expectancies (PAE) were positively associated with alcohol use.
• Negative and positive emotion dysregulation were linked to greater alcohol use.
• Positive, but not negative, emotion dysregulation moderates the link between PAE and use.
• Positive emotion dysregulation may be a risk factor for hazardous substance use in college students who endorse PAE.
• Interventions should address regulation of both negative and positive emotions.

## 1 Introduction

Around half (49.3 %) of college students in the United States (U.S.) endorse drinking alcohol in the past month, with over a quarter of those students (27.4 %) engaging in hazardous alcohol consumption during that timeframe (Substance Abuse and Mental Health Services Administration [SAMHSA], 2021). Given the negative outcomes associated with hazardous drinking in university settings (i.e., unintentional injury, assault, academic problems, health problems, and even death; National Institute on Alcohol Abuse and Alcoholism [NIAAA], 2023), a plethora of resources have been allocated to reducing hazardous drinking in U.S. college students (NIAAA, 2019). Current interventions aimed to reduce hazardous drinking among U.S. college students frequently emphasize skills training and self-monitoring as effective, low-cost intervention targets at the individual level (Carey et al., 2007, Gass et al., 2021, National Institute on Alcohol Abuse and Alcoholism, 2019, Protogerou et al., 2020). These intervention targets often include positive alcohol expectancies and poor negative emotional control, two factors linked to increased hazardous drinking in samples of college students (Brislin et al., 2020, Khan et al., 2018, Lannoy et al., 2021, Ramirez et al., 2020, Weiss et al., 2022, Buckner et al., 2021). Though these interventions are linked with short-term reductions in hazardous alcohol use (Labbe and Maisto, 2011, Scott-Sheldon et al., 2012), hazardous alcohol consumption in college student samples remains high (NIAAA, 2019). Emerging evidence suggests that *positive* emotion dysregulation may be an effective intervention target to reduce hazardous alcohol use, in that it may impact the relationship between positive alcohol expectancies and alcohol use in ways distinct from negative emotion dysregulation (Cho et al., 2019, Paulus et al., 2021, Ramirez et al., 2020, Tovmasyan et al., 2022). However, to date, the role of positive emotion dysregulation in hazardous alcohol use remains largely unexplored.

### 1.1 Alcohol expectancies and alcohol use

Alcohol expectancies, or an individual’s beliefs about the outcomes of drinking, are strongly associated with alcohol consumption behaviors in college aged samples (Crisafulli et al., 2024, Ham and Hope, 2003, McBride et al., 2014, Stamates et al., 2024). Expectancy theories of substance use posit that individuals who believe that alcohol will facilitate a positive outcome are more motivated to consume alcohol (Ramirez et al., 2020, Scott-Sheldon et al., 2012). These motives are typically conceptualized within two dimensions: approach and avoidance motivation, the former of which is aimed at achieving a positive outcome whereas the latter is focused on avoiding a negative outcome (Gius and Schlauch, 2021, Noyes and Schlauch, 2018, Sjödin et al., 2021). Individuals who endorse approach motivation (e.g., those who endorse alcohol use for social enhancement) and those who endorse avoidance motivation (e.g., those who drink to avoid negative emotion) are similarly motivated by positive alcohol expectancies in that they both perceive alcohol to be associated with positive outcomes (Blume & Guttu, 2015). Within college student samples, factor analyses of alcohol expectancy questionnaires yield two to four factors, often centered around improvements in social behavior/assertiveness and sexual enhancement (Jordan et al., 2019, LaBrie et al., 2011, McBride et al., 2014). Accordingly, interventions within college students often aim to diminish these positive expectancies (Gesualdo and Pinquart, 2021, Labbe and Maisto, 2011, Scott-Sheldon et al., 2012). However, though reductions in hazardous drinking are often found (within group effect sizes ranging from 0.13 to 0.36; Scott-Sheldon et al., 2012), studies are often limited by inadequate power to detect intervention effects and sometimes yield equivocal findings regarding reductions in alcohol consumption (Labbe & Maisto, 2011). Given the mixed findings from these interventions, it is likely that they may benefit by jointly considering these positive motives for alcohol use and their interactions with other clinically relevant risk factors for engaging in hazardous drinking, particularly, difficulties in regulating emotion.

### 1.2 Emotion dysregulation and alcohol use

While expectancy theories have traditionally been used to explain the regulation of negative mood as an anticipated positive outcome of alcohol use, recent data suggests that individuals may also engage in alcohol use to regulate positive emotion (Weiss et al., 2015, Weiss et al., 2015, 2018, 2022). In samples of college students, positive emotion regulation is endorsed at both extremes, in that substances may be used because of their anticipated ability to both elicit positive affect (Cooper et al., 2016) and dampen heightened positive emotion (Feldman et al., 2008, Gottfredson and Hussong, 2013). These findings suggest that affect regulation motives for alcohol use may be particularly salient to college students who endorse difficulties regulating positive emotions, as an individual who believes that alcohol will elicit or dampen positive affect and has difficulty with positive emotion dysregulation may be more likely to engage in hazardous drinking to regulate positive affect in contrast to individuals who are able to more effectively regulate positive emotion (Birch et al., 2004, Gautreau et al., 2015, Obasi et al., 2016). Despite findings that emotion regulation may serve as a positive alcohol expectancy in college students (Arbeau et al., 2011, Armeli et al., 2014, Khan et al., 2018), research on the relationship between positive alcohol expectancies and emotion regulation, including an examination of differences in the strength of this relationship based on emotion valence, remains limited. The few studies that exist yield equivocal findings, with two studies finding no significant associations between positive alcohol expectancies and neither positive nor negative emotion regulation (Da Silva, 2019, Dora et al., 2023) and another finding significant correlations of positive alcohol expectancies with negative and positive emotion dysregulation, except difficulties engaging in goal-directed behaviors when experiencing positive emotions (Paulus et al., 2021). Further research is needed to clarify the relationships between alcohol expectancies and both positive and negative emotion regulation.

There exists a small body of literature focused on examining the associations between positive emotion regulation and alcohol use (e.g., Cooper et al., 2016, Emery et al., 2024, Feldman et al., 2008, Gottfredson and Hussong, 2013, Weiss et al., 2018), and these data support the continued investigation of positive emotion dysregulation as a novel risk factor for hazardous alcohol use. The relationship between positive emotion dysregulation and hazardous alcohol use may be particularly relevant to college students, as positive affect dysregulation may play a more salient role in the development, rather than the maintenance, of alcohol misuse (Baker et al., 2004). Alcohol use that serves to positively reinforce positive emotional experiences is commonly endorsed in college samples and has been linked to increases in substance-related problems (Cooper et al., 2016, Howard et al., 2024, Merrill and Read, 2010). Notably, using a brief version of the Difficulties in Emotion Regulation Scale (DERS; Gratz & Roemer, 2004) and the DERS-Positive (Weiss et al., 2015, Weiss et al., 2015)—the former of which focuses on the dysregulation of negative emotions and the latter of which focuses on dysregulation of positive emotions—Paulus and colleagues (2021) found that difficulties with positive emotion regulation demonstrated explanatory value in alcohol consumption above and beyond difficulties regulating negative emotions in college students reporting hazardous drinking. Given these findings, positive emotion dysregulation may be an important treatment target in reducing hazardous alcohol use among college students. However, given the dearth of research regarding the regulation of positive emotion, continued research is needed to elucidate its impact on alcohol use behaviors.

### 1.3 Study rationale and aims

The interaction between positive alcohol expectancies and poor emotional control has been implicated as a risk factor for hazardous alcohol use in emerging adults (Brislin et al., 2020, Lannoy et al., 2021). Research, to date, has focused on the dysregulation of negative emotions and their link to risky alcohol use (see a *meta*-analysis by Weiss et al., 2022 for a review of the literature). However, data suggests that dysregulation of *positive* emotions may also be a salient risk factor for alcohol misuse initiation in developmentally sensitive periods, such as emerging adulthood (Baker et al., 2004, Emery et al., 2024). Thus, the current study aims to clarify the extent to which positive emotion dysregulation and negative emotion dysregulation independently impact the relationship between positive alcohol expectancies and alcohol use in a sample of college students. We hypothesize that 1) positive alcohol expectancies will be positively associated with alcohol use, 2) difficulties in regulating both positive and negative emotions will be independently associated with greater alcohol use, 3) individuals who report concurrently greater positive alcohol expectancies and difficulties in negative emotion regulation will endorse greater hazardous alcohol use when compared to those who report lower positive alcohol expectancies, fewer difficulties with negative emotion regulation, or both, and that, 4) individuals who report concurrently greater positive alcohol expectancies and difficulties in positive emotion regulation will endorse greater hazardous alcohol use when compared to those who report lower positive alcohol expectancies, fewer difficulties with positive emotion regulation, or both.

## 2 Methods

### 2.1 Participants

Participants (N = 165) were undergraduate students, aged 18 to 25 (Mage = 20.48; SDage = 1.90), who were enrolled in a larger scale study of substance use in college students. Inclusion criteria included: being enrolled as an undergraduate student, being between 18 to 25 years of age, and reporting using cannabis and/or alcohol as least three times in the past week. The majority of the sample self-identified as female (66.1 %), with the remaining self-identifying as male. Most participants self-identified as non-Hispanic/Latine (65.5 %) and White (66.7 %), though 11.5 % self-identified as Asian, 12.7 % self-identified as Multiracial, 6.7 % self-identified as Black, 1.8 % self-identified as Native American, and 0.6 % identified as Native Hawaiian or Pacific Islander. There was a near-even distribution across undergraduate level.

### 2.2 Procedures

The current study includes secondary analyses of a larger scale study of substance use in emerging adults enrolled in college. All procedures were approved by the Institutional Review Board at the authors’ university. Participants were recruited via flyer posting and online postings on the university announcement system during the fall and spring semesters of 2023. Interested participants were directed to a secure REDCap database to complete a brief eligibility screening. The brief eligibility survey inquired about individual’s age, undergraduate level, and past week alcohol and marijuana use. Individuals met eligibility criteria for the broader study if they were aged 18–25, currently enrolled as an undergraduate in the university and endorsed more than occasional alcohol and/or marijuana use (i.e., ≥3 times in the previous week; adapted from a prior study (Shircliff et al., under review) and suggestions from NIAAA’s (2024) definitions for hazardous alcohol consumption). Individuals who did not meet eligibility criteria were thanked for their time but were not directed to complete the survey. In total, 51 individuals were deemed ineligible to participate in the study (i.e., 6 individuals denied any alcohol or marijuana use in the past week, 17 endorsed substance use once in the past week, and 28 individuals endorsed substance use 2 times in the past week). Those who met eligibility criteria were directed to an online informed consent form. After consent was acquired, participants completed the 10-minute survey remotely. Participants were compensated $10 for their participation and provided a list of local mental health resources.

### 2.3 Measures

*2.3.1. Positive alcohol expectancies* were measured via the Patient reported outcomes Measurement Information system (PROMIS) alcohol use positive expectancies 7-item short form (Pilkonis et al., 2013). The measure items query attitudes towards positive mental (i.e., “drinking improves a person’s mood.”) and social (i.e. “People have more fun at social occasions when they drink.”) expectancies of alcohol use. Items are rated on a 5-point Likert-type scale ranging from 1 (Not at all) to 5 (Very much) and summed to create a total score; higher scores indicate greater endorsement of positive alcohol expectancies. The measure has previously been validated for use in alcohol using adult samples (Pilkonis et al., 2016) and demonstrated good internal consistency in the present study (Chronbach’s alpha = 0.84)

2.3.2. *Alcohol use* was measured using the PROMIS alcohol use Short-Form (Pilkonis et al., 2013). The first statement, a screening question, is a yes or no question asking: “In the past 30 days, did you drink any type of alcoholic beverage?” if participants answered yes, they are prompted to answer a series of 7 questions about their quantity of use and problems with controlling their consumption and craving (e.g., “I spent too much time drinking”; “It was difficult to get the thought of drinking out of my mind”). Items are scored on a 1 (never) to 5 (almost always) scale and summed together for a total score. Sum scores of 21 and 32 are indicative of borderline significant and clinically significant problems with alcohol use, respectively. The measure demonstrated good internal consistency in the present study (Chronbach’s alpha = 0.89)

2.3.3. Comorbid cannabis use was measured via the *cannabis use Disorders Identification test* (CUDIT; Adamson & Sellman, 2003). The first statement, a screening question, is a *Yes* or *no* question asking: “Have you ever used cannabis over the past 6 months?” if the respondent answers yes, they are then prompted to answer a series of 8 questions. The first question, “How often do you use cannabis?” has response options of 0 (*Never*), 1 (*Monthly or Less*), 2 (*2*–*4 times a Month*), 3 (*2*–*3 times a Week*) and 4 (4 *+ times a Week*). The second question is “How many hours were you “stoned” on a typical day when you had been using cannabis?” The response scale ranges from 0 to 4, with options of 0 (*Less than 1*), 1 (*1 or 2*), 2 (*3 or 4*), 3 (*5 or 6*), 4 (*7 or more*) as answer choices. For questions 3–7, the response scale ranges from 0 to 4, with options of 0 (*Never*), 1 (*Less than Monthly*), 2 (*Monthly*), 3 (*Weekly*), and 4 (*Daily or Almost Daily*) as answer choices. Finally for question 8, the scale includes response options of 0 (*Never*), 2 (*Yes, but not in the past 6 Months*), and 4 (*Yes, during the past 6 Months*). Questions 1–7 are scored on a 0–4 scale and Question 8 is scored 0, 2, or 4 points. Scores of 8 or more are indicative of hazardous cannabis use, and scores of 12 or more can be indicative of a cannabis use disorder. The measure demonstrated excellent internal consistency in the present study (Chronbach’s alpha = 0.90)

2.3.4. *Difficulties with negative emotion regulation* were measured via the difficulties with emotion regulation Scale-Short form (DERS-18; Victor & Klonsky, 2016). The scale consists of 18 items assessing how participants respond to their own emotions (e.g., “When I’m upset, i have difficulty controlling my behaviors.”). Participants are asked to respond to items indicating the frequency at which they endorse these experiences via a 5-point Likert-type scale from 1 (almost never (0–10 %)) to 5 (almost always (91–100 %)). Items are reverse scored where necessary, and their scores are summed to create an overall score. Higher scores indicate greater difficulty regulating negative emotions. The DERS-18 has previously been validated for use in samples of college students, with alphas ranging from 0.79 to 0.92 (Victor & Klonsky, 2016) and demonstrated good internal consistency in the present study (Chronbach’s alpha = 0.87)

2.3.5. *Difficulties with positive emotion regulation* were measured via the difficulties with emotion regulation scale – Positive affect (DERS-PA; Weiss et al., 2015, Weiss et al., 2015). The scale consists of 13 items assessing how participants respond to their own positive emotions and has previously been validated for use in samples of college students (Weiss et al., 2015, Weiss et al., 2015). Items begin with the statement “When I’m happy…” and are completed with different reactions to reflect different dimensions of emotion regulation, including tolerance of positive emotions, engagement in goal-directed behavior, and impulse control during positive emotion states (i.e. “When I’m happy, i feel out of control.”). Participants are asked to respond to items indicating the frequency at which they endorse these experiences via a 5-point Likert-type scale from 1 (almost never (0–10 %)) to 5 (almost always (91–100 %)); responses are summed, with higher scores indicating greater difficulty regulating positive emotion. The measure demonstrated excellent internal consistency in the present study (Chronbach’s alpha = 0.94)

2.3.6 *Psychiatric Comorbities* were measured via two questionnaires. First, anxiety symptoms were measured via the generalized anxiety Disorder-7 (GAD-7; Spitzer et al., 2006), a 7-item questionnaire that asks participants to rank how often they were bothered by anxiety-related problems within a 2-week range. Responses are recorded on a 4-point Likert scale (0 = not at all, 1 = several days, 2 = more than half the days, 3 = nearly every day) and added up to create an overall score. A sum score of 0–4 is indicative of normal or minimal anxiety symptoms, whereas scores of 5–9, 10–14, 15–19, and 20 + are indicative of mild, moderate, moderately severe, and severe anxiety symptoms, respectively. The measure demonstrated adequate internal consistency in the present study (Chronbach’s alpha = 0.81). Second, depressive symptoms were measured via the Patient health Questionnaire-8 (PHQ-8; Kroenke et al., 2009), an 8-item questionnaire that asks participants to rank how often they were bothered by health-related problems (e.g. Feeling tired or having little energy) within a 2-week range. Responses are recorded on a 4-point Likert scale (0 = not at all, 1 = several days, 2 = more than half the days, 3 = nearly every day) and summed to create an overall score. A sum score of 0–4 is indicative of normal or minimal depressive symptoms, whereas scores of 5–9, 10–14, 15–19, and 20 + are indicative of mild, moderate, moderately severe, and severe depressive symptoms, respectively (Kroenke et al., 2009). The measure demonstrated adequate internal consistency in the present study (Chronbach’s alpha = 0.84).

### 2.4 Data analysis plan

All analyses were conducted via SPSS v. 29.0 and the PROCESS Macro extension (Hayes, 2022). Using G*Power 3.1 (Faul et al., 2007), an a priori power analysis was conducted and indicated that a minimum sample size of 98 was necessary to achieve 80 % power for detecting a medium effect of 0.15 at a significance criterion of 0.05 with six predictors (i.e., the main effect of alcohol expectancies (1 in each model, for a total of 2 predictors), the main effect of positive emotion dysregulation, the main effect of negative emotion dysregulation, the interaction between alcohol expectancies and positive emotion dysregulation, the interaction between alcohol expectancies and negative emotion dysregulation). Next, descriptive statistics were conducted of the full sample and key study variables (i.e., positive alcohol expectancies, negative emotion dysregulation, positive emotion dysregulation, and alcohol use). Bivariate correlations were then conducted to examine the associations between all key study variables. No collected demographic variables were significantly associated with alcohol use (i.e., age, gender, race, ethnicity), therefore no demographic covariates were included in the models. To determine the unique contributions of positive versus negative affect dysregulation, we included regulation of the opposite affect as a covariate in each model. All statistical assumptions were met (i.e., linearity, independence of errors, homoscedasticity, multivariate normality, little multicollinearity). Multiple linear regression analyses with moderation were then conducted. Separate models were run for each moderator (i.e., negative and positive emotion regulation variables). Post hoc-analyses were also run to adjust for hazardous cannabis use and psychiatric comorbidities (i.e., generalized anxiety and depressive symptoms).

## 3 Results

### 3.1 Alcohol use

The majority of the sample (93.8 %) endorsed alcohol use in the previous 30 days. Notably, the remaining 6.2 % of the sample met inclusion criteria for the larger study through endorsement of cannabis use, but not alcohol use, within the past 30 days. About 17 % of the sample (n = 28) were at the minimum threshold for borderline significant problems with alcohol use, and 4.8 % (n = 8) were deemed as having clinically significant problems with alcohol use.

### 3.2 Correlations among key study variables

Alcohol use shared a moderate, positive association with positive alcohol expectancies, difficulties with negative emotion regulation, and difficulties with positive emotion regulation. Difficulties with positive emotion regulation shared a moderate positive association with difficulties with negative emotion regulation. Cannabis use was not significantly associated with any study variables. Depressive symptoms shared a moderate positive correlation with alcohol use and difficulties with positive emotion regulation, and a strong positive correlation with difficulties with negative emotion regulation. Anxiety symptoms shared a moderate positive correlation with alcohol use and difficulties with positive emotion regulation, and a strong positive correlation with difficulties with negative emotion regulation and depressive symptoms (Table 1).

**Table 1:** Correlations, Means, and standard deviations of study variables.

Variable	1	2	3	4	5	6	7
1. Positive alcohol expectancies	−						
2. Alcohol use	0.31** (<0.001) N = 160	−					
3. Difficulties with negative emotion regulation	0.13 (0.10) N = 154	0.31** (<0.001) N = 1155	−				
4. Difficulties with positive emotion regulation	0.02 (0.85) N = 160	0.27* (0.001) N = 161	0.47** (<0.001) N = 155	−			
5. Cannabis use	−0.08 (0.27) N = 160	0.03 (0.69) N = 161	0.04 (0.59) N = 155	0.06 (0.46) N = 161	−		
6. Depressive symptoms	0.06 (0.50) N = 160	0.31** (<0.001) N = 161	0.63** (<0.001) N = 155	0.40** (<0.001) N = 161	0.13 (0.09) N = 161	−	
7. Anxiety symptoms	0.02 (0.80) N = 159	0.36** (<0.001) N = 160	0.73** (<0.001) N = 154	0.36** (<0.001) N = 160	0.15 (0.07) N = 160	0.77** (<0.001) N = 160	−
Mean	23.80	15.98	2.52	0.12	8.10	16.25	14.54
Standard Deviation	5.33	7.23	0.69	0.05	7.89	5.21	5.54

*Note.* * indicates *p* = 0.001.

** indicates *p <* 0.001.

### 3.3 Moderation analyses

**Fig. 1:**
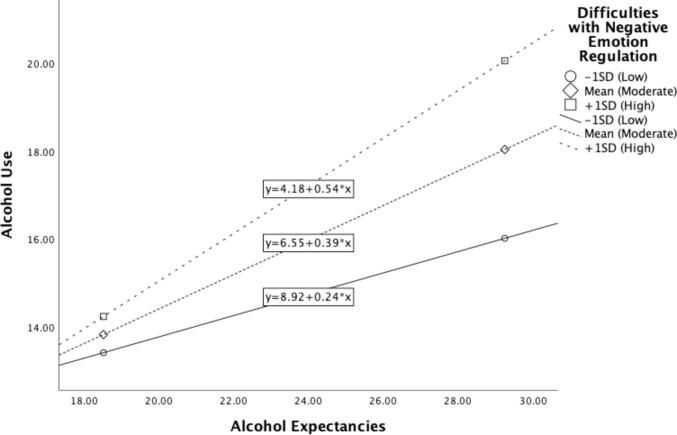
The relationship between positive alcohol expectancies and alcohol use does not change significantly based on the level of difficulty with negative emotion regulation.

Multiple linear regression was conducted to assess whether difficulties regulating negative emotion moderated the relationship between positive alcohol expectancies and alcohol use, while adjusting for the effect of positive emotion dysregulation on alcohol use. The overall regression model was statistically significant (R^2^ = 0.20, F (4, 149) = 9.32, p < 0.001). Neither positive alcohol expectancies (β = -0.16, p > 0.05) nor difficulties with negative emotion regulation (β = -3.42, p > 0.05) were significantly associated with greater alcohol use. Difficulties with negative emotion regulation did not significantly moderate the relationship between positive alcohol expectancies and alcohol use after accounting for the effect of positive emotion dysregulation (β = 0.22, p > 0.05; Table 2; Fig. 1).

**Table 2:** Summary of moderation analysis predicting hazardous alcohol use (N = 154).

Variable	*B*	*SE*	*t*	*p*
Positive alcohol expectancies	−0.16	0.38	−0.41	0.68
Difficulties with negative emotion regulation	−3.42	3.76	−0.91	0.37
Interaction	0.22	0.15	1.47	0.15
**Difficulties with positive emotion regulation**	**23.73**	**10.91**	**2.17**	**0.03**

*Note.* Bold indicates < 0.05.

**Fig. 2:**
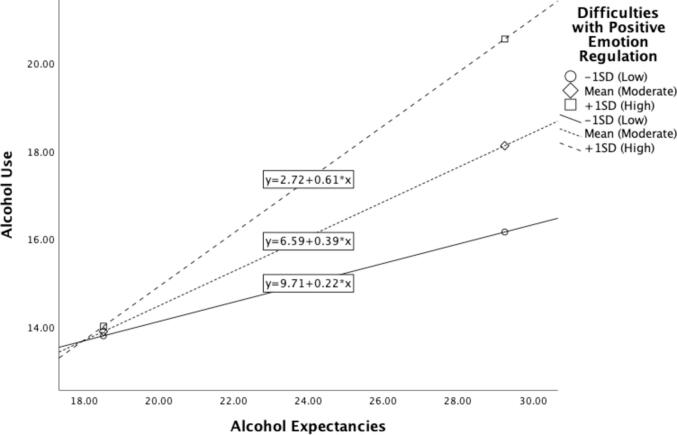
The relationship between positive alcohol expectancies and alcohol use changes significantly based on the level of difficulty with positive emotion regulation.

Next, multiple linear regression was conducted to test if difficulties regulating positive emotion moderated the relationship between positive alcohol expectancies and alcohol use while adjusting for the effects of negative emotion dysregulation on alcohol use. The overall regression model was statistically significant (R^2^ = 0.21, F (4, 149) = 9.97, p < 0.001). Neither positive alcohol expectancies (β = -0.08, p > 0.05) nor difficulties with positive emotion regulation (β = -69.51, p > 0.05) were independently associated with greater alcohol use. However, difficulties with positive emotion regulation moderated the relationship between positive alcohol expectancies and alcohol use after accounting for the effect of negative emotion dysregulation (β = 3.87, p = 0.034). Specifically, college students who reported greater positive alcohol expectancies and concurrently greater difficulties with positive emotion regulation also reported greater alcohol consumption, compared to those who reported greater positive alcohol expectancies and fewer difficulties regulating positive emotion (Table 3). The relationship between positive alcohol expectancies and alcohol use was significant at high (β = 0.61, p < 0.001) and moderate (β = 0.40, p = 0.001) levels of difficulties with positive emotion regulation, but not at low levels (β = 0.22, p > 0.05; Fig. 2).

**Table 3:** Summary of moderation analysis predicting hazardous alcohol use (N = 154).

Variable	*B*	*SE*	*t*	*p*
Positive alcohol expectancies	−0.08	0.25	−0.31	0.75
Difficulties with positive emotion regulation	−69.51	45.27	−1.53	0.13
**Interaction**	**3.87**	**1.87**	**2.07**	**0.04**
**Difficulties with negative emotion regulation**	**2.03**	**0.87**	**2.34**	**0.02**

*Note.* Bold indicates < 0.05.

### 3.4 Post-hoc moderation analyses with psychiatric comorbidities as covariates

Multiple linear regression was conducted to assess whether difficulties regulating negative emotion moderated the relationship between positive alcohol expectancies and alcohol use, while adjusting for the effect of positive emotion dysregulation, depressive symptoms, and anxiety symptoms on alcohol use. The overall regression model was statistically significant (R^2^ = 0.26, F (6, 146) = 8.36, p < 0.001). Neither positive alcohol expectancies (β = -0.08, p > 0.05) nor difficulties with negative emotion regulation (β = -5.59, p > 0.05) were significantly associated with greater alcohol use. Difficulties with negative emotion regulation did not significantly moderate the relationship between positive alcohol expectancies and alcohol use after accounting for the effect of positive emotion dysregulation and mental health comorbidities (β = 0.19, p > 0.05; Table 4).

**Table 4:** Summary of post-hoc moderation analysis predicting hazardous alcohol use (N = 153).

Variable	*B*	*SE*	*t*	*p*
Positive alcohol expectancies	−0.08	0.38	−0.21	0.83
Difficulties with negative emotion regulation	−5.59	3.73	−1.49	0.14
Interaction	0.19	0.14	1.38	0.17
Difficulties with positive emotion regulation	21.91	11.22	1.95	0.05
Depressive symptoms	0.03	0.17	0.20	0.84
**Anxiety Symptoms**	**0.43**	**0.17**	**2.59**	**0.01**

*Note.* Bold indicates < 0.05.

Multiple linear regression was conducted to assess whether difficulties regulating positive emotion moderated the relationship between positive alcohol expectancies and alcohol use, while adjusting for the effect of negative emotion dysregulation, depressive symptoms, and anxiety symptoms on alcohol use. The overall regression model was statistically significant (R^2^ = 0.26, F (6, 146) = 8.41, p < 0.001). Neither positive alcohol expectancies (β = 0.09, p > 0.05) nor difficulties with positive emotion regulation (β = -45.21, p > 0.05) were significantly associated with greater alcohol use. Difficulties with positive emotion regulation did not significantly moderate the relationship between positive alcohol expectancies and alcohol use after accounting for the effect of negative emotion dysregulation and mental health comorbidities (β = 2.74, p > 0.05; Table 5).

**Table 5:** Summary of post-hoc moderation analysis predicting hazardous alcohol use (N = 153).

Variable	*B*	*SE*	*t*	*p*
Positive alcohol expectancies	0.09	0.25	0.35	0.73
Difficulties with positive emotion regulation	−45.21	45.93	−0.98	0.33
Interaction	2.74	1.88	1.46	0.15
Difficulties with negative emotion regulation	−0.41	1.19	−0.34	0.73
Depressive symptoms	0.04	0.17	0.25	0.80
**Anxiety Symptoms**	**0.39**	**0.17**	**2.32**	**0.02**

*Note.* Bold indicates < 0.05.

### 3.5 Post-hoc moderation analyses with hazardous cannabis use as covariates

Multiple linear regression was conducted to assess whether difficulties regulating negative emotion moderated the relationship between positive alcohol expectancies and alcohol use, while adjusting for the effect of positive emotion dysregulation and hazardous cannabis use on alcohol use. The overall regression model was statistically significant (R^2^ = 0.20, F (5, 148) = 7.45, p < 0.001). Neither positive alcohol expectancies (β = -0.17, p > 0.05) nor difficulties with negative emotion regulation (β = -3.52, p > 0.05) were significantly associated with greater alcohol use. Difficulties with negative emotion regulation did not significantly moderate the relationship between positive alcohol expectancies and alcohol use after accounting for the effect of positive emotion dysregulation and cannabis use (β = 0.22, p > 0.05). Cannabis use did not serve as a significant covariate within the model (β = -0.03, p > 0.05; Table 6).

**Table 6:** Summary of post-hoc moderation analysis predicting hazardous alcohol use (N = 154).

Variable	*B*	*SE*	*t*	*p*
Positive alcohol expectancies	−0.17	0.38	−0.45	0.65
Difficulties with negative emotion regulation	−3.52	3.78	−0.93	0.35
Interaction	0.22	0.15	1.49	0.14
Cannabis Use	−0.03	0.07	−0.44	0.67
**Difficulties with positive emotion regulation**	**23.96**	**10.95**	**2.19**	**0.03**

Note. Bold indicates < 0.05.

Multiple linear regression was conducted to assess whether difficulties regulating positive emotion moderated the relationship between positive alcohol expectancies and alcohol use, while adjusting for the effect of negative emotion dysregulation and hazardous cannabis use on alcohol use. The overall regression model was statistically significant (R^2^ = 0.21, F (5, 148) = 7.98, p < 0.001). Neither positive alcohol expectancies (β = -0.09, p > 0.05) nor difficulties with positive emotion regulation (β = -70.99, p > 0.05) were significantly associated with greater alcohol use. Difficulties with positive emotion regulation significantly moderated the relationship between positive alcohol expectancies and alcohol use after accounting for the effect of negative emotion dysregulation and cannabis use (β = 3.94, p = 0.04). Cannabis use did not serve as a significant covariate within the model (β = -0.03, p > 0.05; Table 7).

**Table 7:** Summary of post-hoc moderation analysis predicting hazardous alcohol use (N = 154).

Variable	*B*	*SE*	*t*	*p*
Positive alcohol expectancies	−0.09	0.25	−0.37	0.71
Difficulties with positive emotion regulation	−70.99	45.48	−1.56	0.12
**Interaction**	**3.94**	**1.88**	**2.09**	**0.04**
Cannabis Use	−0.03	0.07	−0.49	0.62
**Difficulties with negative emotion regulation**	**2.05**	**0.88**	**2.35**	**0.02**

Note. Bold indicates < 0.05.

## 4 Discussion

The current study examined the independent moderating roles of negative and positive emotion dysregulation on the association between positive alcohol expectancies and alcohol use. Our findings suggest that greater alcohol expectancies, greater difficulty regulating positive emotion, and greater difficulty regulating negative emotion shared a small to moderate positive association with alcohol use. Notably, difficulties regulating positive emotion, but not negative emotion, moderated the relationship between alcohol expectancies and alcohol use, even after accounting for the influence of the opposite emotion valence. Specifically, college students who reported greater positive alcohol expectancies and concurrently greater difficulties with positive emotion regulation also reported greater alcohol consumption, compared to those who reported greater positive alcohol expectancies and fewer difficulties regulating positive emotion, even after accounting for the impact of negative emotion dysregulation. The research and clinical implications of these findings are discussed below.

Neither positive alcohol expectancies nor difficulties with negative emotion regulation independently predicted alcohol use, and their combined effect was nonsignificant. Though previous work has demonstrated insignificant associations between these variables and alcohol use (Da Silva, 2019, Dora et al., 2023), these findings were contrary to our hypotheses, as previous research largely links positive alcohol expectancies (Ham and Hope, 2003, McBride et al., 2014, Crisafulli et al., 2024, Stamates et al., 2024) and negative emotion dysregulation with increases in alcohol consumption (Buckner et al., 2021, Miller and Racine, 2022, Veilleux et al., 2014, Weiss et al., 2022). These findings may be due to the context in which our sample used substances, as some literature supports that alcohol expectancies are context-specific, and differentially predict drinking behaviors across settings (Hurd et al., 2020, Wolkowicz et al., 2019). For example, for individuals who use alcohol in situations in which positive expectancies and positive emotion regulation may be more relevant (e.g., social gatherings or parties), the ability to regulate negative emotions may be less influential on alcohol consumption when compared to situations in which alcohol is consumed to cope with stress or negative emotions (Ham et al., 2011, Lac and Luk, 2019, LaRowe et al., 2021, Studer et al., 2014). It may also be that positive alcohol expectancies in general do not interact with negative emotion regulation. Instead, specific reward expectancies related to a reduction in negative affect (e.g., tension reduction, positive changes in experience, cognitive reappraisal), rather than those related to eliciting or heightening positive affect (e.g., sexual enhancement, social enhancement, or arousal), may be more relevant to negative emotion regulation. Future research may benefit from evaluating how these discrete items within positive alcohol expectancy measures interact with emotion regulation skills to predict alcohol use. For example, measure items that query the attenuation of negative emotions as a positive alcohol expectancy (e.g., “drinking improves a person’s mood”) versus those centered on increasing positive emotions (e.g., “people have more fun at social occasions when they drink”) may differentially interact with positive and negative emotion dysregulation, and these effects may not be found when using an overall score reflecting the frequency at which these positive alcohol expectancies are endorsed. Examining how discrete items within expectancy measures interact with negative versus positive emotional dysregulation could allow researchers to better characterize the nature of positive alcohol expectancies to pinpoint the specific reward expectancies that are most relevant to different emotion regulation difficulties, thereby offering more targeted insights for interventions.

Findings suggest a significant (though diminutive) synergistic effect exists between positive alcohol expectancies and difficulties with positive emotion regulation. Particularly, individuals exhibiting higher positive alcohol expectancies coupled with greater difficulties with positive emotion regulation demonstrated amplified alcohol use, when compared to individuals who reported positive alcohol expectancies and fewer difficulties with positive emotion dysregulation. This interaction suggests that the combined influence of positive alcohol expectancies and deficits in positive emotion regulation may reinforce each other, exacerbating the propensity for heightened alcohol consumption behaviors beyond the effects of each factor independently. These findings are in line with existing data suggesting positive emotion dysregulation may be a risk factor for individuals who drink to enhance positive emotions (Birch et al., 2004; Gautrau, 2015; Obasi et al., 2016). It may be that samples of college students are more likely to endorse enhancement expectancies than coping expectancies for alcohol use, thus making positive emotion dysregulation a more salient risk factor for hazardous alcohol use in this particular group. Arbeau and colleages (2011) found that when college students experienced heightened positive affect, they were more likely to report higher enhancement expectancies for alcohol use. Notably, these findings highlighting alcohol’s link with positive affect regulation are in line with Peele and Grant’s (1999) psychosocial model of substance use. The model underscores the role of habitual, moderate use, of alcohol in facilitating psychosocial well-being for individuals who consume alcohol to prolong or incite pleasurable experiences. Indeed, previous research suggests that college students may use substances because of their anticipated ability to both elicit positive affect (Cooper et al., 2016) and dampen heightened positive emotion (Feldman et al., 2008, Gottfredson and Hussong, 2013). By recognizing the psychosocial benefits that some individuals associate with habitual alcohol use, substance use interventions can more effectively address the underlying motivations for drinking. Together, these findings suggest that college students may have a greater inclination to consume alcohol to intensify or prolong positive emotions, and these motivations may synergistically interact with difficulties regulating positive emotion to increase risk for hazardous substance use. Interventions may benefit from tailoring to individual motivations and emotion regulation skills, particularly through helping college students identify surges in positive emotions and reducing desires to amplify those emotions through alcohol consumption.

Post-hoc analyses indicated that hazardous cannabis use did not serve as a significant covariate in the model, however, analyses that adjusted for psychiatric comorbidities diminished the effects of prior findings, such that the interaction between positive alcohol expectancies and difficulties with positive emotion regulation was no longer significant. Anxiety symptoms served as a particularly important covariate, in that they may contribute to both heightened emotional dysregulation and more pronounced alcohol expectancies (Corran, 2020, Crisafulli et al., 2024, Paulus et al., 2017). Given that both positive and negative emotion dysregulation are proposed as transdiagnostic factors contributing to anxiety (Hofmann et al., 2012, Suveg et al., 2010), and emotion dysregulation has been supported as a mediator for the relationship between anxiety and hazardous alcohol use (Sorid et al., 2021), it could be that anxiety symptomology masks the specific influence of positive alcohol expectancies on both negative and positive emotion dysregulation, thereby diminishing the observed interaction between the two variables. These findings that anxiety, not depressive, symptoms serve as a significant covariate are in line with findings that college students who use substances have significantly higher levels of anxiety, but not depressive, symptoms than those who did not use substances (Prosek et al., 2018) and that anxiety, but not depressive, symptoms are related to alcohol use problems among young adults (Howell et al., 2010). Continued research is needed to effectively parse apart the unique role of positive emotion dysregulation from anxiety symptomology in order to develop more targeted interventions for college students engaging in hazardous alcohol use.

This study is not without limitations. First, we cannot attribute causality to the associations between alcohol expectancies, emotion regulation, and alcohol use due to the cross-sectional nature of the study. Second, no objective measures of substance use were used, and data may be subject to recall bias and response bias across measures. Third, the inclusion criteria for the larger project focused on the frequency of alcohol use (i.e., three or more times per week), rather than the quantity consumed per occasion. Given these limitations, it may be that our sample reflects both hazardous alcohol use and individuals at-risk for hazardous alcohol use given their more frequent use of substances, rather than only hazardous substance users. Fourth, we focused on intrapersonal factors (i.e., emotion dysregulation) that were hypothesized to impact the relationship between alcohol expectancies and alcohol use. It is likely there are various interpersonal factors (e.g., alcohol use as a means of socialization) that also impact this relationship. Fifth, we did not collect data on use of substances other than alcohol and cannabis use. Therefore, our findings on the impact of emotion regulation may not translate to the use of other substances, such as opioid, methamphetamine, or benzodiazepine use and misuse. Lastly, the representativeness of the sample may be limited, as the sample consisted mostly of White, non-Hispanic, college students. Additional research is needed to determine whether findings replicate within a more diverse, community sample to understand how cultural and sociodemographic factors may impact alcohol expectancies and use.

Despite these limitations, the current study suggests that difficulties with positive emotion regulation serves as a risk factor for hazardous substance use in college students who endorse positive alcohol expectancies. The study addressed a current gap in the literature by examining the independent effects of positive and negative emotion dysregulation in a sample of at-risk college students. Generally, findings suggest that both positive and negative emotion dysregulation are associated with increases in alcohol consumption. Moreover, difficulties with positive emotion regulation further exacerbates the relationship between positive alcohol expectancies and alcohol use above that of negative affect dysregulation. Most research on the relationship between emotion regulation and substance use focuses on negative emotion; in line with shifting literature (e.g., Cooper et al., 2016, Emery et al., 2024, Feldman et al., 2008, Gottfredson and Hussong, 2013, Weiss et al., 2018), our findings suggest that difficulties with positive emotions should be included in future studies to understand how positive and negative emotion influence college students’ decisions to engage in hazardous alcohol use. Similarly, clinical interventions should be shifted to consider perceived positive outcomes of habitual alcohol use and to teach college students effective strategies to manage not only negative emotion but heightened positive emotion as well. In research and clinical work with college students, it is crucial to identify how difficulties with emotion regulation may confer risk for hazardous alcohol use across time while also accounting for various interpersonal factors that have been linked to alcohol use; doing so may reduce risk for hazardous alcohol use and associated negative outcomes for this high-risk population.

This work was supported by Texas Tech University.

Data Availability Statement: The data that support the findings of this study are available from the corresponding author, CC, upon reasonable request.

## CRediT authorship contribution statement

**Katherine Shircliff:** Writing – review & editing, Writing – original draft, Formal analysis, Conceptualization. **Haley Coronado:** Writing – review & editing, Writing – original draft. **Madeline McClinchie:** Writing – review & editing, Writing – original draft. **Caroline Cummings:** Supervision, Software, Resources, Project administration, Methodology, Investigation, Funding acquisition, Data curation.

## Declaration of competing interest

The authors declare that they have no known competing financial interests or personal relationships that could have appeared to influence the work reported in this paper.

## Data Availability

Data will be made available on request.
